# From across the Seas

**Published:** 1917-05-19

**Authors:** 


### FROM ACROSS THE SEAS.

### Municipal Control of Hospitals.

IN Australia there is a suggestion, supported by the Local Government Association, for hospital reform in the direction of the hospitals being placed under the control of the municipalities. Minutes just to hand from the Sydney Municipal Council show that that authority does not consider that they would be beneficial.

### Medical Charities at the Cape.

Two new institutions have been established under the Cape Hospital Board, whose report for 1916-17 was recently issued; these are the McGregor Convalescent Home (mainly for children) and the Salt River Free Dispensary. The Board is now responsible for the management and financing of five hospitals, of which the chief is Somerset Hospital, Cape Town, two convalescent homes, two free dispensaries, and one district nursing organisation. The total cost of the maintenance of this important group during 1916 amounted to £85,919. Only £5,488 of the sum thus required was produced by voluntary contributions, collected by the individual institutions, while the patients themselves contributed towards the cost of maintenance £8,973, and the State and local authorities provided £35,097. Allocations from the Board's own income, amounting to £34,852, made up the greater part of the balance required. From the admirably arranged tables of patients' statistics we learn that 4,322 patients were treated in 1916, 686 were cared for at the convalescent homes, over 25,000 visits to the dispensaries were made, and nurses attended 561 patients in their own homes.

### News from New Zealand.

THE Auckland Hospital Board is not in accord with the findings of the Wellington Conference reported in our issue of April 14, and views with disfavour the suggestions that the requirements of the hospitals in the Dominion should be " pooled " at some central distributing store, and that inspectors be appointed to supervise the collection of fees for hospital treatment. The board, in addition, strongly protests against any legislative authority being given to the Minister of Public Health, as suggested by him, to enable the Government to tighten control over the expenditure of hospital boards.

The Auckland Board has also had a difference of opinion with the Auditor-General, who objects to the payment of half-salary to Dr. A. M. Grant, recently acting-medical superintendent, who is now absent on active service. The Auditor-General requires that the money thus paid must be refunded within twenty-one days, and that if this is not done the members of the board will be surcharged under the Public Revenues Act, 1910. The defence of the board is that they were asked by Government only temporarily to fill vacant medical posts while the war lasts, and that Dr. Grant's original post of junior resident medical officer was open to him on his return. Such Government control as exists in New Zealand is evidently a very live thing.

